# Multiple tools to investigate the origin of the exotic species Chinook salmon *Oncorhynchus tshawytscha* (Walbaum, 1792) (Salmonidae) in the world's largest chocked coastal lagoon

**DOI:** 10.1111/jfb.70151

**Published:** 2025-07-20

**Authors:** Yan Gonçalves Gowert, Valéria Marques Lemos, Fabiano Corrêa, Sabrina Vollrath, João Paes Vieira, Mario Vinicius Condini, Rodrigo Ferreira Bastos, Mylla Carla Cescon Freire, Ana Paula Cazerta Farro, Antonio Sergio Varela Junior, Cristiano Albuquerque, Maurício Hostim‐Silva, Alexandre Miranda Garcia

**Affiliations:** ^1^ Laboratório de Ictiologia, Universidade Federal do Rio Grande Rio Grande Brazil; ^2^ Laboratório de Genética e Biologia Molecular, Universidade Estadual do Maranhão Caxias Brazil; ^3^ Laboratório de Ecologia de Peixes Marinhos, Universidade Federal do Espírito Santo São Mateus Brazil; ^4^ Programa de Pós‐Graduação em Oceanografia Ambiental, Universidade Federal do Espírito Santo Vitória Brazil; ^5^ Laboratório de Genética e Conservação Animal, Centro Universitário Norte do Espírito Santo (CEUNES), Universidade Federal do Espírito Santo São Mateus Brazil; ^6^ Laboratório de Morfologia, Universidade Federal do Rio Grande Rio Grande Brazil; ^7^ Departamento de Biociências, Universidade Federal Rural do Semiárido Mossoró Brazil

**Keywords:** aquaculture, biological invasion, ecosystem impact, invasive fish, Patos lagoon

## Abstract

Salmonid species have increasingly been established in South America for aquaculture and recreational fishing purposes. This study provides the first scientific record of the Chinook salmon (*Oncorhynchus tshawytscha*) in the Patos Lagoon (32°S), the world's largest choked coastal lagoon. The specimen was a female with 770 mm (total length), 3992.1 g, estimated age of 6 years and an advanced sexual maturation stage. Based on multiple tools (DNA barcoding, stable isotopes in eye lens, otolith growth layers and egg's histological analysis), we discussed potential entry routes for this species into the lagoon.

Originally distributed throughout the Northern Hemisphere, Salmonidae have increasingly been recorded outside their natural range, particularly in South America, for aquaculture and recreational fishing (Macchi & Vigliano, 2014; Nomura, 1977; Soto et al., 2022; Vigliano et al., 2007). Among introduced salmon species, Chinook salmon (*Oncorhynchus tshawytscha*) (Walbaum, 1792) has shown remarkable adaptability, establishing populations in Patagonia (Ciancio et al., 2015; Figueroa‐Muñoz et al., 2023; Musleh et al., 2020). Its introduction began in 1924 with egg imports from the United States released into rivers in the Los Lagos region of Chile (Soto et al., 2007). In the 1970s, ocean farming started in Chile, and by the 1990s, major escapes were recorded beyond Chilean basins, reaching Patagonia (Fernández et al., 2010; Figueroa‐Muñoz et al., 2023; Liotta, 2019; Soto et al., 2001, 2007). Currently, this species occurs in 48 south American river basins — 32 draining to the Pacific, 16 to the Atlantic — spanning over 3 million km^2^, mainly in Chile and Argentina, with some records in Uruguay (Figueroa‐Muñoz et al., 2023) and other continents worldwide (Correa & Gross, 2008; Crawford & Muir, 2007).

Chinook salmon are anadromous, native to the Pacific Ocean (Taylor, 1990). In its natural habitats, they hatch in freshwater, migrate to the ocean to mature, and return to natal rivers to spawn and die (Healey, 1991; Quinn, 2018). Juveniles from some populations migrate to the ocean the same year (ocean type), others after a year in freshwater (stream type) (DuBois & Liller, 2010; Healey, 1991; Murphy et al., 2013).

This study provides the first scientific record of *O. tshawytscha* in the Patos Lagoon (32° S), southern Brazil, the world's largest (~10,000 km^2^) choked coastal lagoon (Kjerfve, 1986). Multiple tools (DNA barcoding, stable isotopes in eye lens, otolith growth layers and histological analysis of eggs) were used to investigate possible entry routes into the lagoon system.

The Patos Lagoon provides conditions for spawning, growth, feeding and refuge for numerous fish species. Approximately 80% of the lagoon's is limnic, whereas its estuarine zone corresponds to ~10%, with a permanent ocean connection (Figure 1). The area hosts diverse habitats (wetlands, lakes, rivers) (Seeliger, 2001). Moreover, there are numerous sites cultivating exotic species around the lagoon's margin and its drainage basin (Troca et al., 2012; Troca & Vieira, 2012). In recent years, the number of exotic species in the lagoon has been increasing significantly (Bertaco et al., 2022; Cardoso et al., 2025; Chuctaya et al., 2019; Fontoura et al., 2016; Garcia et al., 2004; Gowert et al., 2025; Saccol‐Pereira et al., 2006).

**FIGURE 1:**
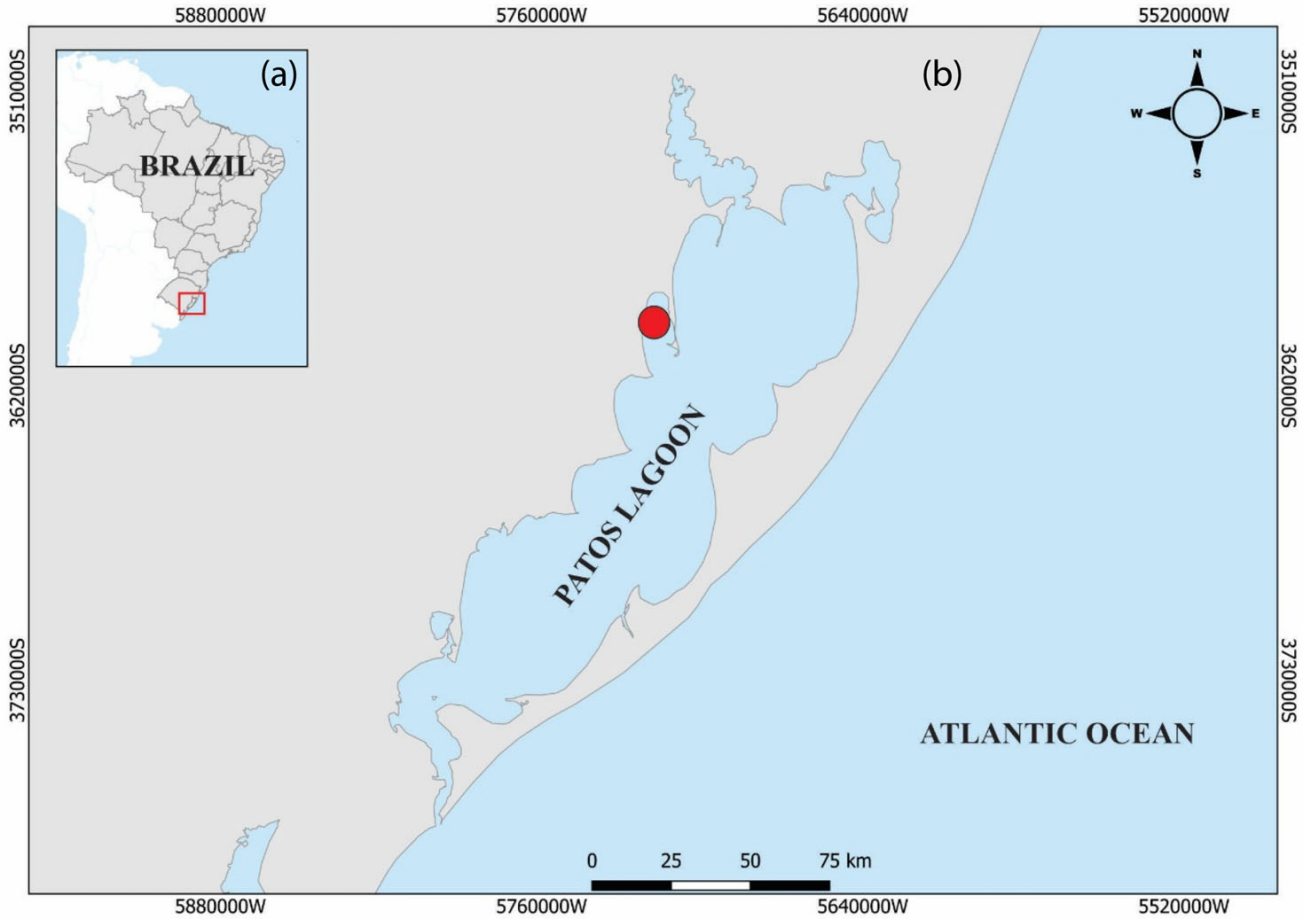
(a) Map showing the location of Patos Lagoon in Brazil. (b) Area where the specimen of *Oncorhynchus tshawytscha* (represented by the red circle) was caught inside Patos Lagoon.

The salmonid was caught on 4 October 2023, by local fishermen in northern reaches (30°45′45″ S, 51°25′13″ W) of Patos Lagoon (Figure 1). The individual, though partialy eviscerated by the fishermen, had gonad remnants that allowed reproductive analysis. The specimen was frozen, measured, weighed and sampled for DNA barcoding. Additional analysis included otolith age estimation and stable isotopes in the eye lens to infer its life history. The specimen was deposited at the scientific collection of the Ichthyology Laboratory at Federal University of Rio Grande (FURG) (CIFURG #2961).

Molecular analyses were performed at the Animal Genetics and Conservation Laboratory, Federal University of Espírito Santo. Genomic DNA was extracted using a saline protocol (Bruford et al., 1992) and diluted to 20 ng/μL. Four tissue replicates were processed. A fragment of the cytochrome oxidase subunit I (COI) gene was amplified using primers FishF1 and FishR2 (Ward et al., 2005). PCRs used 20‐μL reactions with standard reagents and thermal cycles. Fragments were visualized on 1% agarose gel stained with Gelred®. Reactions were purified using 1.8 μL of the enzyme ExoSap‐IT. Purified PCR products were sequenced in both directions (forward and reverse) by an outsourced company.

Sequences were edited and aligned in MEGA 7.026 (Kumar et al., 2016) and compared to GenBank and BOLD databases. The degree of homology provides a percentage that varies between 0% to 100% (higher values mean greater genetic proximity between the sequences compared). A dataset with 50 COI sequences from *Salmo* and *Oncorhynchus* species was compiled for phylogenetic analysis via a neighbour‐joining tree (Table S1) (Saitou & Nei, 1987) using the Kimura‐2 model (Kimura, 1980), with *Betta splendens* Regan, 1910 as outgroup. Branch confidence was tested with 10,000 bootstrap replicates (Felsenstein, 1985) (Figure S1).

To facilitate visualization of the specimen analysed in this study and to have greater reliability in the analysis, we chose to use five sequences per species, when available, from different published articles to generate the neighbour‐joining tree. The specimen was included in the GenBank as code PV715700.

Sagital otoliths were extracted, embedded in resin and sectioned (0.4 mm) using a diamond saw. Slices were polished and photographed under transmitted light. Annual growth bands were identified independently by three readers following methods used for *Oncorhynchus mykiss* (Walbaum 1792) (Hining et al., 2000).

The specimen's eyes were extracted, and because no significant isotopic differences are expected between the right and left lenses (Wallace et al., 2014; Young et al., 2022), the right lenses were used to collect samples from laminae formed throughout ontogeny. When further delamination was not feasible due to the required sample size, the innermost remaining sample was designated as the ‘core’, or the earlier ontogeny sample considered (sensu Bastos et al., 2024). The samples were placed directly into tin capsules and oven‐dried for 12 h at 60°C. These samples were analysed at the Integrated Analysis Center of the Federal University of Rio Grande. The stable isotopes of carbon (^13^C/^12^C) and nitrogen (^15^N/^14^N) elements were measured using continuous flow elemental analyser isotope ratio mass spectrometry. Certified standards were run between every 12 samples and compared to the international reference standards for C and N (Vienna Pee Dee Belemnite and atmospheric N_2_, respectively). Replicate precision was 0.28‰ (^13^C) and 0.07‰ (^15^N).

Gonad remnants were processed to make histological slides using the protocol of Beçak and Paulete (1970) and analysed under an optical microscope using the Brown‐Peterson et al. (2011) maturation scale. Oocyte diameters were measured with ImageJ software at the FURG Morphology Laboratory.

Our results based on multiple investigative tools provided not only an accurate taxonomic identification at species level of the captured salmonid but also further ecological evidence to investigate plausible entry routes for this specimen into the lagoon (Figure 2). All sequences evaluated showed between 99.7% and 100% similarity with sequences of the *O. tshawytscha* species in both databases. In the Neighbor‐Joining Kimura tree, the sequence clustered in a high‐support clade specific to the species (Figure S1), corroborating the results of the similarity index made with the GenBank and BOLD databases. The specimen measured 770 mm (length) and weighed 3992.1 g (see Table S2 for additional morphometric and meristic measurements). Otoliths indicated an age of 6 years old (Figure 2a). Due to autosamples issues, some eye lens layers were lost, but δ^13^C ranged from −25.0 ‰ to −16.0 ‰ and δ^15^N from 5.0 ‰ to 22.0 ‰ (Figure 2c). Histological analysis revealed a female with oocytes in complete vitellogenesis (average cell diameter 2.83 ± 0.28 mm) (Figure 2d).

**FIGURE 2:**
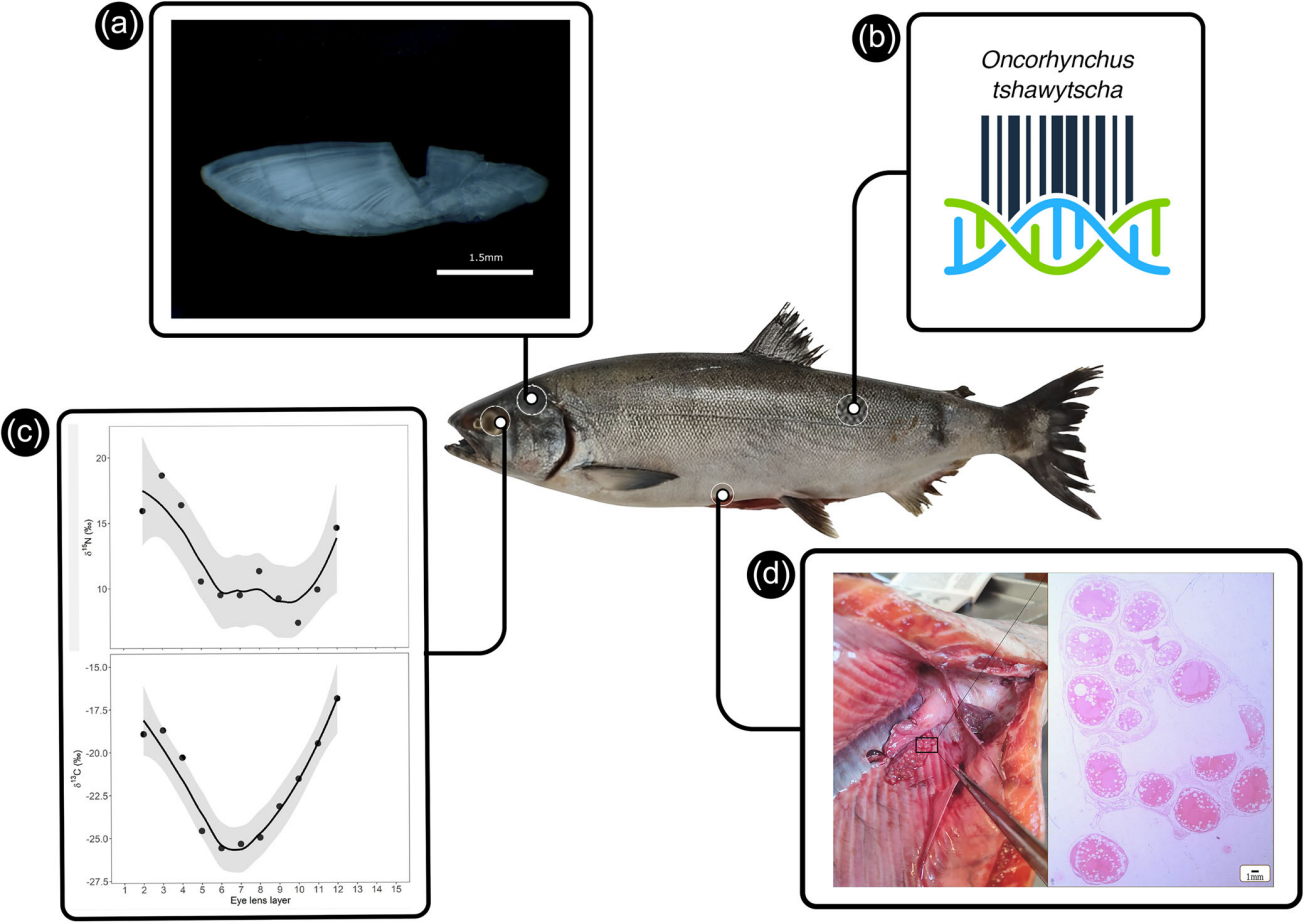
The specimen of *Oncorhynchus tshawytscha* captured in the Patos Lagoon, Brazil. The results of multiple tissues and tools used to investigate its biological and ecological features: (a) otolith showing growth bands, scale bar 1.5 mm and (b) barcoding analysis to species identification (see Table S1 and Figure 2 for results). (c) Laminae isotopic values from the salmon eye lens. The upper panel shows the δ^15^N values while the lower panel shows the δ^13^C values. Locally estimated scatterplot smoothing (loess) curve in black with a shaded grey area representing the confidence interval (95%) showing the general trend along ontogeny. (d) Histological section of its gonads showing high frequency of oocytes (haematoxylin–eosin). Scale bar 1 mm.

To our knowledge, our work provides the first scientific record of the Chinook salmon *O. tshawytscha* at the Patos Lagoon (32°S) in southern Brazil. It is worth noting that Figueroa‐Muñoz and collaborators (2023) mentioned its occurrence in this lagoon as the northernmost record for this species in the Atlantic Ocean. However, they do not provide any information supporting this affirmation, such as site location (latitude/longitude), time of the capture, biometric data of the specimen or in which scientific collection the specimen was deposited. In fact, they provide information only for specimens collected in South American countries adjacent to south Brazil, such as Uruguay, Paraguay and Argentina and in the Paraná drainage basin. In contrast, our study site (the Patos Lagoon) belongs to a different drainage basin (~200.000 km^2^) distributed along the east and south portion of the southernmost state (Rio Grande do Sul) in Brazil and the northeastern portion of Uruguay (Asmus, 1997). Hence, it seems reasonable to assume that there was a misinterpretation by Figueroa‐Muñoz and collaborators (2023) of the geographical location of the Patos Lagoon (Lagoa dos Patos in Portuguese) in south Brazil, with the Laguna Los Patos in Chile, which appears in some of the cited works by them (Correa & Gross, 2008; Soto et al., 2007).

Initially, we formulated three hypotheses regarding the probable origins of the *O. tshawytscha* specimen presented here. First, it could come from aquaculture tanks around the Patos Lagoon basin. However, this hypothesis was discarded as 14 fish species are cultivated in the region and salmonoids are not among them (Troca, 2013). The second hypothesis suggests this fish could have invaded the Patos Lagoon after continental displacement through naturally or artificially interconnected waterways between Chile, Argentina and Brazil. This idea was already suggested to explain the occurrence of other exotic species in the Patos Lagoon (Bertaco & Azevedo, 2023; Pinto et al., 2000). Although Chinook salmon escapes from Argentinean and Chilean salmon farms have already occurred (Grosman, 1992; Soto et al., 2001), the lack of their records in the Uruguay river significantly reduces the possibility of continental displacement, making this hypothesis less plausible. The third hypothesis considers that the fish entered the Patos Lagoon through the estuarine route as suggested for Argentina (Macchi & Vigliano, 2014), which aligns with the natural migratory pattern of certain salmon species. According to this idea, our captured individual may have originated from past generations that escaped an oceanic farming system, probably in Chile. They were then displaced across Patagonia through a system connected to the Atlantic Ocean, such as the Santa Cruz Basin, eventually reaching the northern Argentine coastal waters and finally arriving at the mouth of the Patos Lagoon estuary. This hypothesis gains support from the fact that *O. tshawytscha* ocean farming began in the 1970s, leading to massive escapes beyond the Chilean basins and reaching the Patagonia region, where it spawned in many areas (Soto et al., 2001). It was estimated that more than 3 million salmon escaped from rearing facilities located in the inland seas of southern Chile during the 1990s. Among these individuals, Chinook salmon represented a significant proportion, with the number of escapees reaching 50,000 individuals (Soto et al., 2001).

The results of the carbon (δ^13^C) and nitrogen (δ^15^N) stable isotopes of the eye lenses and otolith growth increments may also help to support this third hypothesis. First, isotopic composition in the eye lens suggests early‐life oceanic support, as the sustained decrease in δ^13^C values and increase in δ^15^N from older to more recent‐formed layers indicate a marine isotopic baseline during the initial ontogenetic stages (Coletto et al., 2021; Maruyama et al., 2023; Troina et al., 2020). Subsequently, a rapid decrease in both δ^13^C and δ^15^N implies no residence time in the estuarine area of the Patos Lagoon because the typical isotopic baseline values in the region fall within an intermediate range (Claudino et al., 2013; Garcia et al., 2016; Possamai et al., 2020). Conversely, the lower values measured in our specimen's tissue align with fish inhabiting the freshwater reaches of the Patos Lagoon (where it was caught), which are characterized by lower stable isotopic values (especially δ^13^C) due to the prevalence of plants using the C3 photosynthetic pathway in this region (Garcia et al., 2007; Mont'Alverne et al., 2016). Second, salmon growth is a multifaceted process influenced by various biotic and abiotic factors (Kaeriyama et al., 2000; Kelley, 2001; Wells et al., 2008), where growth rates tend to be higher in marine habitats compared to fresh water (Gross, 1987; Vøllestad et al., 2004). Also, Chinook salmon exhibit increased marine growth rates during periods of elevated sea surface temperatures (Myers et al., 2010). Given that we estimated a growth rate consistent with that expected for a 6‐year‐old salmon raised under favourable growth conditions (Bigler et al., 1996; Healey, 1991; Quinn, 2018), this seems to provide additional support that our sampled specimen used the marine pathway to move into the Patos Lagoon system.

Finally, it is worth highlighting that the habitat heterogeneity and abundant food resources found at the Patos Lagoon and its estuarine zone may be favourable for the development and establishment of *O. tshawytscha* populations. Considering the sampled salmon specimen had gonads in an advanced maturation stage, we may be facing the establishment of a future Chinook salmon population in the region. This invasion could threaten native species and the lagoon's ecological functions and ecosystem services in the near future.

## AUTHOR CONTRIBUTIONS

All authors made significant contributions to the development of this work. Yan Gonçalves Gowert, Valéria Marques Lemos and Alexandre Miranda Garcia were responsible for data collection, sample processing in the laboratory, data analysis, literature review and drafting the initial version of the manuscript. Fabiano Corrêa, Sabrina Vollrath, João Paes Vieira, Mário Vinícius Condini, Rodrigo Ferreira Bastos, Mylla Cescon, Ana Paula Cazerta Farro, Antônio Sérgio Varela Júnior, Maurício Hostim‐Silva and Cristiano Albuquerque contributed to data acquisition (DNA barcoding, stable isotopes from eye lenses, otolith growth readings, egg histological analysis) and data analysis, and conducted a thorough review of the manuscript.

## Supporting information


**FIGURE S1.** Neighbour‐joining tree based on the Kimura 2‐parameter model with 10,000 repetitions for the 11 species of the Salmonidae family analysed in this study. The numbers in each branch indicate the bootstrap values, the number in front of the species refers to the GenBank accession number and the sample in blue is the specimen identified in this study.

**TABLE S1.** GenBank and BOLD sequences used in this study. Species name, database used, access number and reference.

**TABLE S2.** Morphometric and meristic measurements of salmon.
