# Supplementary material for: Multiple tools to investigate the origin of the exotic species Chinook salmon Oncorhynchus tshawytscha (Walbaum, 1792) (Salmonidae) in the world's largest chocked coastal lagoon
Source: J Fish Biol. 2025 Jul 20;107(5):1800–6. doi: 10.1111/jfb.70151 (PMC12710837; doi:10.1111/jfb.70151)
Supplement: Supplementary file 1 — FIGURE S1. Neighbour‐joining tree based on the Kimura 2‐parameter model with 10,000 repetitions for the 11 species of the Salmonidae family analysed in this study. The numbers in each branch indicate the bootstrap values, the number in front of the species refers to the GenBank accession number and the sample in blue is the specimen identified in this study. [file JFB-107-1800-s001.docx]

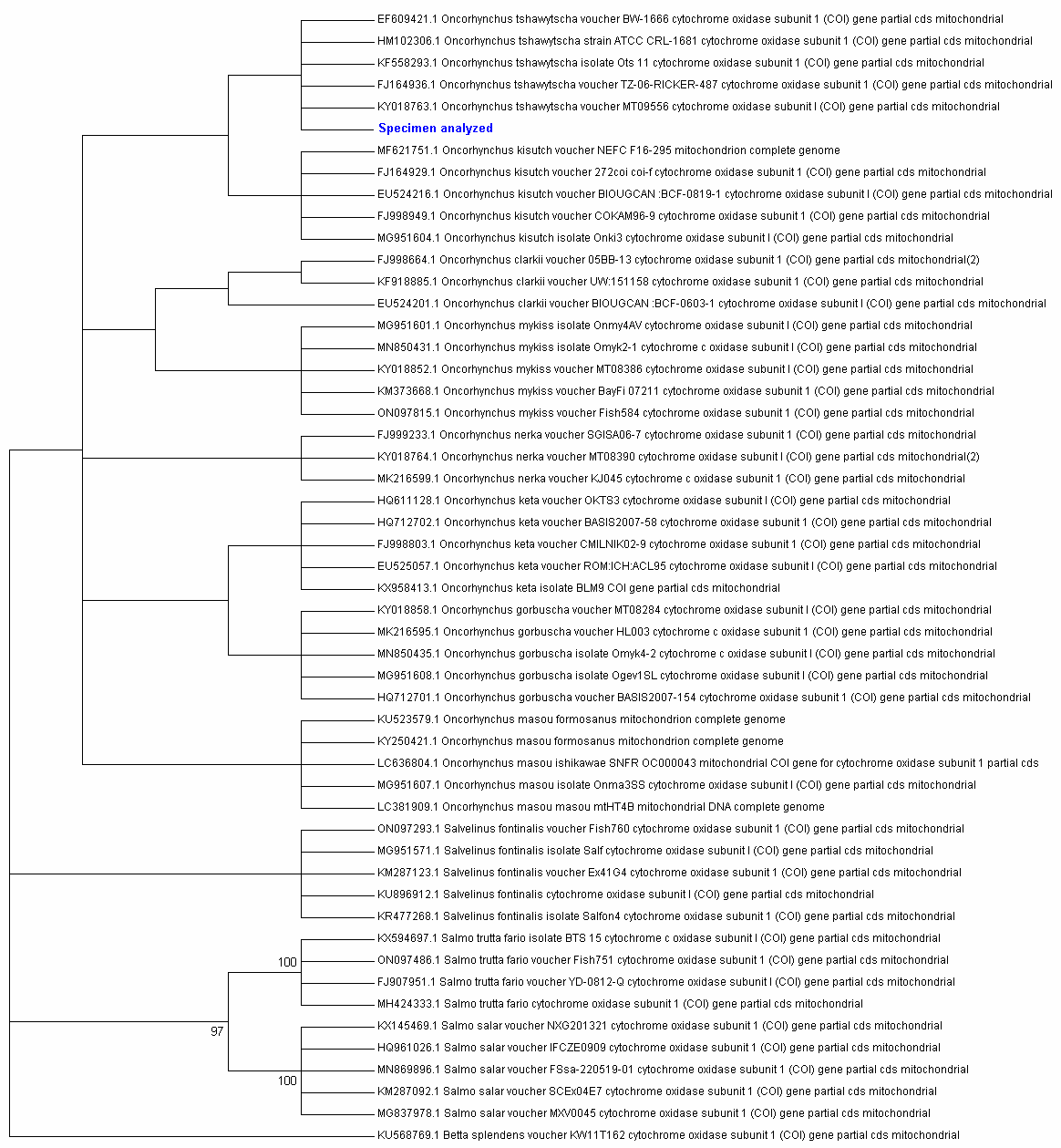


Supplementary Figure 1: Neighbor-Joining tree based on the Kimura 2-parameter model with 10.000 repetitions for the 11 species of the Salmonidae family analyzed in this study. The numbers in each branch indicate the Bootstrap values, the number in front the species refers to the GenBank accession number and the sample in blue is the specimen identified in this study.
