# Supplementary material for: Multiple tools to investigate the origin of the exotic species Chinook salmon Oncorhynchus tshawytscha (Walbaum, 1792) (Salmonidae) in the world's largest chocked coastal lagoon
Source: J Fish Biol. 2025 Jul 20;107(5):1800–6. doi: 10.1111/jfb.70151 (PMC12710837; doi:10.1111/jfb.70151)
Supplement: Supplementary file 2 — TABLE S1. GenBank and BOLD sequences used in this study. Species name, database used, access number and reference. [file JFB-107-1800-s003.docx]

**Supplementary Material**

**Table S1** GenBank and Bold sequences used in this study. Species name, database used, access number and reference

| **Specie** | **Database** | **Access number** | **Reference** |
| --- | --- | --- | --- |
| *Oncorhynchus clarkii* | GenBank | FJ998664.1 | Rasmussen et al., 2009 |
| *Oncorhynchus clarkii* | GenBank | EU524201.1 | Hubert et al., 2008 |
| *Oncorhynchus clarkii* | GenBank | FJ998663.1 | Rasmussen et al., 2009 |
| *Oncorhynchus clarkii* | GenBank | FJ998662.1 | Rasmussen et al., 2009 |
| *Oncorhynchus chrysogaster* | *GenBank* | JX960908.1 | Crete-Lafrenier et al., 2012 |
| *Oncorhynchus gilae apache* | GenBank | JX960907.1 | Crete-Lafrenier et al., 2012 |
| *Oncorhynchus gorbuscha* | GenBank | HQ712701.1 | Mecklenburg et al., 2011 |
| *Oncorhynchus gorbuscha* | GenBank | MG951608.1 | Artamonova et al., 2018 |
| *Oncorhynchus gorbuscha* | GenBank | KY018858.1 | Guenther et al., 2017 |
| *Oncorhynchus gorbuscha* | GenBank | MN850435.1 | Xiong et al., 2020 |
| *Oncorhynchus gorbuscha* | GenBank | MK216595.1 | Chen et al., 2019 |
| *Oncorhynchus keta* | GenBank | HQ712702.1 | Mecklenburg et al., 2011 |
| *Oncorhynchus keta* | GenBank | HQ611128.1 | Cawthorn et al., 2011 |
| *Oncorhynchus keta* | GenBank | FJ998803.1 | Rasmussen et al., 2009 |
| *Oncorhynchus keta* | GenBank | EU525057.1 | Hubert et al., 2008 |
| *Oncorhynchus keta* | GenBank | HQ611127.1 | Cawthorn et al., 2011 |
| *Oncorhynchus kisutch* | GenBank | FJ164929.1 | Steinke et al., 2009 |
| *Oncorhynchus kisutch* | GenBank | MG951604.1 | Artamonova et al., 2018 |
| *Oncorhynchus kisutch* | GenBank | FJ998949.1 | Rasmussen et al., 2009 |
| *Oncorhynchus kisutch* | GenBank | EU524216.1 | Hubert et al., 2008 |
| *Oncorhynchus kisutch* | GenBank | MF621751.1 | Schroeter et al., 2020 |
| *Oncorhynchus masou* | GenBank | MG951607.1 | Artamonova et al., 2018 |
| *Oncorhynchus masou* | GenBank | LC381909.1 | Kato-Unoki et al., 2020 |
| *Oncorhynchus masou* | GenBank | KU523579.1 | Ho et al., 2016 |
| *Oncorhynchus masou* | GenBank | MG951606.1 | Artamonova et al., 2018 |
| *Oncorhynchus masou* | GenBank | MN850433.1 | Xiong et al., 2020 |
| *Oncorhynchus mykiss* | GenBank | ON097815.1 | Zangl et al., 2022 |
| *Oncorhynchus mykiss* | GenBank | KM373668.1 | Knebelsberger et al., 2015 |
| *Oncorhynchus mykiss* | GenBank | MG951601.1 | Artamonova et al., 2018 |
| *Oncorhynchus mykiss* | GenBank | KY018852.1 | Guenther et al., 2017 |
| *Oncorhynchus mykiss* | GenBank | MN850431.1 | Xiong et al., 2020 |
| *Oncorhynchus nerka* | GenBank | KY018764.1 | Guenther et al., 2017 |
| *Oncorhynchus nerka* | GenBank | MK216599.1 | Chen et al., 2019 |
| *Oncorhynchus nerka* | GenBank | FJ999233.1 | Rasmussen et al., 2009 |
| *Oncorhynchus tshawytscha* | GenBank | KY018763.1 | Guenther et al., 2017 |
| *Oncorhynchus tshawytscha* | GenBank | FJ164936.1 | Steinke et al., 2009 |
| *Oncorhynchus tshawytscha* | GenBank | KF558293.1 | Brandl et al., 2015 |
| *Oncorhynchus tshawytscha* | GenBank | EF609421.1 | Ward et al., 2007 |
| *Oncorhynchus tshawytscha* | GenBank | HM102306.1 | Cooper et al., 2007 |
| *Salmo trutta fario* | GenBank | MH424333.1 | Kalayci et al., 2018 |
| *Salmo trutta fario* | GenBank | ON097485.1 | Zangl et al., 2022 |
| *Salmo trutta fario* | GenBank | ON097680.1 | Zangl et al., 2022 |
| *Salmo trutta fario* | GenBank | ON097486.1 | Zangl et al., 2022 |
| *Salmo salar* | GenBank | MG837978.1 | Sarmiento-Camacho et al., 2018 |
| *Salmo salar* | GenBank | KM287092.1 | Knebelsberger et al., 2015 |
| *Salmo salar* | GenBank | G837977.1 | Sarmiento-Camacho et al., 2018 |
| *Salmo salar* | GenBank | KM287091.1 | Knebelsberger et al., 2015 |
| *Salmo salar* | GenBank | KM287090.1 | Knebelsberger et al., 2015 |
| *Salvelinus fontinalis* | GenBank | ON097293.1 | Zangl et al., 2022 |
| *Salvelinus fontinalis* | GenBank | KM287123.1 | Knebelsberger et al., 2015 |
| *Salvelinus fontinalis* | GenBank | MG951571.1 | Artamonova et al., 2018 |
| *Salvelinus fontinalis* | GenBank | KU896912.1 | Faulks et al., 2016 |
| *Salvelinus fontinalis* | GenBank | KR477268.1 | Thalinger et al., 2016 |

**References**

Artamonova, V. S., Kolmakova, O. V., Kirillova, E. A., & Makrov, A. A. (2018). Phylogeny of Salmonoid Fishes (Salmonoidei) Based on mtDNA COI Gene Sequences (Barcoding). Contemporary Problems of Ecology. 11: 271–285. <https://doi.org/10.1134/S1995425518030022>

Brandl, S., Schumer, G., Schreier, B. M., Conrad, J. L., May, B., & Baerwald, M. R. (2015). Ten real-time PCR assays for detection of fish predation at the community level in the San Francisco Estuary-Delta. Molecular Ecology Resources. 15(2): 278-284. <https://doi.org/10.1111/1755-0998.12305>

Cawthorn, D. M., Steinman, H. A., & Witthuhn, R. C. (2011). Establishment of a mitochondrial DNA sequence database for the identification of fish species commercially available in South Africa. Molecular Ecology Resources. 11(6): 979-91. <https://doi.org/10.1111/j.1755-0998.2011.03039.x>

Chen, K. C., Zakaria, D., Altarawneh, H., Andrews, G. N., Ganesan, G. S., John, K. M., Khan, S., & Ladumor, H. (2019). DNA barcoding of fish species reveals low rate of package mislabeling in Qatar. Genome. 62(2): 69-76. <https://doi.org/10.1139/gen-2018-0101>

Cooper, J. K., Sykes, G., King, S., Cottrill, K., Ivanova, N. V., Hanner, R., & Ikonomi, P. (2007). Species Identification in Cell Culture: A Two-Pronged Molecular Approach. In Vitro Cellular & Developmental Biology. Animal. 43(10): 344–351. <https://doi.org/10.1007/s11626-007-9060-2>

Crête-Lafrenière, A., Weir, L. K., & Bernatchez, L. (2012). Framing the Salmonidae Family phylogenetic Portrait: a more complete Picture from increased táxon sampling. PLoS One. 7(10): e46662. <https://doi.org/10.1371/journal.pone.0046662>

Faulks, L., & Östman, Ö. (2016). Genetic Diversity and Hybridisation between Native and Introduced Salmonidae Fishes in a Swedish Alpine Lake. PLoS One. 11(3): e0152732. <https://doi.org/10.1371/journal.pone.0152732>

Günther, B., Raupach, M. J., & Knebelsberger, T. (2017). Full-length and mini-length DNA barcoding for the identification of seafood commercially traded in Germany. Food Control. 73: 922-929. <https://doi.org/10.1016/j.foodcont.2016.10.016>

Ho, C. W., Chen, J. J. W., Lee, T. H., & Lin, H. J. (2016). Complete mitochondrial genome of *Oncorhynchus masou formosanus* (Jordan & Oshima, 1919) (Pisces, Salmonidae). Mitochondrial DNA B Resour. 1(1): 295-296. <https://doi.org/10.1080/23802359.2016.1166084>

Hubert, N., Hanner, R., Holm, E., Mandrak, N. E., Taylor, E., Burridge, M., Watkinson, D., Dumont, P., Curry, A., Bentzen, P., Zhang, J., April, J., & Bernatchez, L. (2008). Identifying Canadian freshwater fishes through DNA barcodes. PLoS One. 3(6): e2490. <https://doi.org/10.1371/journal.pone.0002490>

Kalayci, G., Ozturk, R. C., Capkin, E., & Altinok, I. (2018). Genetic and molecular evidence that brown trout Salmo trutta belonging to the Danubian lineage are a single biological species. Journal of Fish Biology. 93: 792-804. <https://doi.org/10.1111/jfb.13777>

Kato-Unoki, Y., Umemura, K., & Tashiro, K. (2020). Fingerprinting of hatchery haplotypes and acquisition of genetic information by whole-mitogenome sequencing of masu salmon, *Oncorhynchus masou masou*, in the Kase River system, Japan. PLoS One. 15(11): e0240823. <https://doi.org/10.1371/journal.pone.0240823>

Knebelsberger, T., Dunz, A. R., Neumann, D., & Geiger, M. F. (2015). Molecular diversity of Germany's freshwater fishes and lampreys assessed by DNA barcoding. Molecular Ecology Resources. 15(3): 562-72. <https://doi.org/10.1111/1755-0998.12322>

Mecklenburg, C. W., Moller, P. R., & Steinke, D. (2011). Biodiversity of arctic marine fishes: taxonomy and zoogeography. Marine Biodiversity. 41: 109-140. <https://doi.org/10.1007/s12526-010-0070-z>

Rasmussen, R. S., Morrissey, M. T., & Hebert, P. D. (2009). DNA barcoding of commercially important salmon and trout species (Oncorhynchus and Salmo) from North America. Journal of Agricultural and food chemistry. 57(18): 8379-8385. <https://doi.org/10.1007/s12237-019-00693-0>

Sarmiento-Camacho, S., & Valdez-Moreno, M. (2018). DNA barcode identification of commercial fish sold in Mexican markets. Genome. 61(6): 457-466. <https://doi.org/10.1139/gen-2017-0222>

Schroeter, J., Maloy, A. P., Rees, C. B., & Bartron, M. (2020). Fish mitochondrial genome sequencing: expanding genetic resources to support species detection and biodiversity monitoring using environmental DNA. Conservation Genetics Resources. 12: 433–446. <https://doi.org/10.1007/s12686-019-01111-0>

Steinke, D., Zemlak, T. S., Boutillier, J. A., & Hebert, P. D. N. (2009). DNA barcoding of Pacific Canada’s fishes. Marine Biology. 156: 2641–2647. <https://doi.org/10.1007/s00227-009-1284-0>

Thalinger, B., Oehm, J., Mayr, H., Obwexer, A., Zeisler, C., & Traugott, M. (2016). Molecular prey identification in Central European piscivores. Molecular Ecology Resources. 16(1): 123-37. <https://doi.org/10.1111/1755-0998.12436>

Ward, R. D., & Holmes, B. H. (2007). An analysis of nucleotide and amino acid variability in the barcode region of cytochrome *c* oxidase I (cox1) in fishes.  Molecular Ecology Notes. 7: 899 –907. <https://doi.org/10.1111/j.1471-8286.2007.01886.x>

Xiong, X., Huang, M., Xu, W., Cao, M., Li, Y., & Xiong, X. (2020). Tracing Atlantic Salmon (*Salmo salar*) in Processed Fish Products Using the Novel Loop-Mediated Isothermal Amplification (LAMP) and PCR Assays. Food Anal. Methods. 13, 1235–1245. <https://doi.org/10.1007/s12161-020-01738-y>

Zangl, L., Schäffer, S., Daill, D., Friedrich, T., Gessl, W., Mladinić, M., Sturmbauer, C., Wanzenböck, J., Weiss, S. J., & Koblmüller, S. (2022). A comprehensive DNA barcode inventory of Austria's fish species. PLoS One. 17(6): e0268694. <https://doi.org/10.1371/journal.pone.0268694>
