# Supplementary material for: Multiple tools to investigate the origin of the exotic species Chinook salmon Oncorhynchus tshawytscha (Walbaum, 1792) (Salmonidae) in the world's largest chocked coastal lagoon
Source: J Fish Biol. 2025 Jul 20;107(5):1800–6. doi: 10.1111/jfb.70151 (PMC12710837; doi:10.1111/jfb.70151)
Supplement: Supplementary file 3 — TABLE S2. Morphometric and meristic measurements of salmon. [file JFB-107-1800-s002.docx]

Supplementary Table 2: Morphometric and meristic measurements of salmon

| **Morphometric character** | **measurements (mm)** |
| --- | --- |
| head length | 365 |
| base of the first dorsal fin | 73 |
| base of the adipose fin | 16 |
| length of caudal peduncle | 57 |
| pre-orbital distance | 48 |
| eye diameter | 13 |
| post-orbital distance | 350 |
| maximum body heigth | 180 |
| heigth of the caudal peduncle | 51 |
| pectoral fin length | 106 |
| base of the anal fin | 89 |
| standard length | 640 |
| total length | 770 |
| upper jaw length | 77 |
| head heigth | 129 |
| base of the pectoral fin | 29 |
| pre-dorsal distance | 33 |
| pre-caudal portion (trunk) | 116 |
| caudal portion | 139 |
| caudal fin length | 132 |
| mouth width | 36 |
| interorbital distance | 62 |
| Furciferous length | 730 |

| **meristic character** | **n** |
| --- | --- |
| dorsal fin | 3 + 11 |
| pectoral fin | 15 |
| pelvic fin | 2 + 9 |
| anal fin | 16 |
| lateral line scales | 156 |
| scales dorsal base to lateral line | 25 |
| scales adipose base to lateral line | 15 |
| tracks 1st upper gill arch | 10 |
| tracks 1st lower gill arch | 12 |
